# CD8+ T-Cell Repertoire in Human Leukocyte Antigen Class I-Mismatched Alloreactive Immune Response

**DOI:** 10.3389/fimmu.2020.588741

**Published:** 2021-01-20

**Authors:** Florence Bettens, Zuleika Calderin Sollet, Stéphane Buhler, Jean Villard

**Affiliations:** Transplantation Immunology Unit and National Reference Laboratory for Histocompatibility, Geneva University Hospitals, Geneva, Switzerland

**Keywords:** T-cell alloreactivity, human leukocyte antigen (HLA), T-cell repertoire, T-cell receptor, hematopoietic stem cell transplantation (HSCT)

## Abstract

In transplantation, direct allorecognition is a complex interplay between T-cell receptors (TCR) and HLA molecules and their bound peptides expressed on antigen-presenting cells. In analogy to HLA mismatched hematopoietic stem cell transplantation (HSCT), the TCR CDR3β repertoires of alloreactive cytotoxic CD8^+^ responder T cells, defined by the cell surface expression of CD137 and triggered *in vitro *by HLA mismatched stimulating cells, were analyzed in different HLA class I mismatched combinations. The same HLA mismatched stimulatory cells induced very different repertoires in distinct but HLA identical responders. Likewise, stimulator cells derived from HLA identical donors activated CD8^+^ cells expressing very different repertoires in the same mismatched responder. To mimic *in vivo* inflammation, expression of HLA class l antigens was upregulated *in vitro* on stimulating cells by the inflammatory cytokines TNFα and IFNβ. The repertoires differed whether the same responder cells were stimulated with cells treated or not with both cytokines. In conclusion, the selection and expansion of alloreactive cytotoxic T-cell clonotypes expressing a very diverse repertoire is observed repeatedly despite controlling for HLA disparities and is significantly influenced by the inflammatory status. This makes prediction of alloreactive T-cell repertoires a major challenge in HLA mismatched HSCT.

## Introduction

Solid-organ and hematopoietic stem cell transplantations (HSCT) are characterized by an immune response mediated by direct, indirect and semi-direct T-cell allorecognition (1–5). In the context of HSCT, HLA compatibility between the donor and recipient is critical to prevent severe complications such as graft versus host disease (GVHD). The current standard of HLA compatibility includes the loci HLA-A, B, C, DRB1, and DQB1, with HLA-DPB1 matching considered additionally according to the number of potential compatible donors. Although HLA compatibility (i.e., the so-called 10/10 matching) is the best option, a mismatched situation can also be considered, usually involving one mismatch at a single HLA class I or class II locus (i.e., 9/10) (6–12).

HLA class I molecules have extremely high allelic polymorphism (ebi.ac.uk/ipd/imgt/hla/stats.html) (13), potentially influencing direct alloreactivity in HLA mismatched situations. Indeed, we and others (14–16) have previously shown that HLA alloantigens can induce variable strengths of alloreactive T-cell response in cellular *in vitro* assays and *in vivo*, emphasizing the potential role of the TCR repertoire of the alloreactive responder T-cell population.

The T-cell repertoire is initially shaped in the thymus by positive and negative selection of maturing T cells on self-peptide-HLA complexes and then modulated overtime at the periphery by the cumulative history of foreign antigenic exposures (2–5). Cross-reactivity and flexibility of the T-cell receptor (TCR) allow each TCR to potentially interact with many HLA-peptide complexes (5, 17). While the CDR1 and CDR2 loops of the TCR interact primarily with cognate HLA molecules, the most variable region of the TCR, encoded by the third complementary region (CDR3) of α and β chains, is specifically involved in antigenic peptide recognition. Its nucleotide sequence, generated by somatic rearrangements of V(D)J gene segments and the random insertion/deletion of nucleotides, allows to characterize unique T-cell clonotypes. Powerful high-throughput T-cell receptor sequencing technology has been proposed as an approach to study T-cell response at the clonal level (18).

In HLA mismatched HSCT, TCRs of the donor’s T cells can cross-react with non-self-HLA-peptide complexes expressed on the recipient’s cells and thereby elicit a direct alloreactive immune response, which can induce a strong clinical complication called GVHD (19).

In the context of semi or fully HLA mismatched situations, quantitative analyses of *in vitro* induced alloimmune responses have revealed that up to one-tenth of circulating CD4^+^ and CD8^+^ T-cell clones are potentially alloreactive, accounting thus for the large diversity of the alloresponse (20, 21). In a single HLA-DPB1 mismatched situation, Arrieta-Bolanos et al. (22) demonstrated that alloreactive CD4^+^ T-cell repertoires had virtually no overlapping TCR rearrangements in three different HLA-DPB1*04:02 individuals when stimulated by two different HeLa cells (i.e., expressing either HLA-DPB1*02:01 or DPB1*09:01). The clonal diversity was independent of the level of alloreactivity and was not based on HLA-DPB1 alloantigen structure and dissimilarity between responder and stimulator cells.

In this study, we have investigated the specificity of the alloreactive cytotoxic CD8^+^ T-cell repertoire by using as an *in vitro* model a one-way mixed lymphocyte reaction (MLR) assay. We have performed the analyses on specific HLA class I incompatibilities according to two scenarios. In the first one, T cells derived from a given anonymous blood donor (responder cells) were stimulated with cells from distinct blood donors (stimulator cells) mismatched for the same HLA. In the second one, responder T cells of different HLA-matched anonymous blood donors were stimulated with HLA-mismatched cells from the same given blood donor. This experimental approach investigates the specific cytotoxic CD8^+^ T-cell response in a more physiological environment involving other leukocytes like helper T cells and monocytes, representing an approximation of events occurring during the *in vivo* direct alloreactive immune response. In addition, to mimic the effect of inflammation induced by a clinical event such as infection or conditioning regimen, HLA molecules expressed by stimulator cells were upregulated by transiently incubating cells with the inflammatory cytokines, namely tumor necrosis factor alpha (TNFα) and interferon beta (IFNβ) (23).

## Materials and Methods

### Cells

Peripheral blood mononuclear cells (PBMCs) were purified using standard Ficoll procedure from blood collected from anonymous donors who have been HLA genotyped at loci A, B, C, DRB1, DRB3/4/5, DQB1 and DPB1 at high resolution by the Swiss National Reference Laboratory for Histocompatibility (LNRH), while searching potential unrelated HSC donors. Cells were cryopreserved in RPMI 1640 medium (Gibco, Life Technologies, Oslo Norway) supplemented with 10 mM l-glutamine, 100 units/ml, penicillin/streptomycin (Gibco), 10% heat-inactivated human AB serum (own preparation) and 10% DMSO (Merk, Darmstadt, Germany). HLA typing was performed by reverse PCR-sequence-specific oligonucleotide microbeads arrays and high throughput sequencing (One Lambda, Canoga Park, USA). Unstimulated total CD8^+^ T cells (average purity of 95.8%) were isolated from PBMCs by negative selection using a CD8 cell magnetic microbeads isolation kit (No. 130-096-495) (Miltenyi Biotec, Bergisch Gladbach, Germany).

### Mixed Lymphocyte Reactions

One way MLRs were performed as previously described (14, 24). Briefly, responder PBMC cells (2x10^6^) were stimulated at a ratio of 1:1 with 30Gy irradiated stimulator PBMC cells in RPMI 1640 medium (Gibco) supplemented with 10 mM l-glutamine, 100 units/ml penicillin/streptomycin (Gibco) and 10% human AB serum (own preparation). Twenty units per milliliter rIL-2 (Peprotech, London, UK) were added at days 3, 7, and 11. After 13 days of culture, responding T cells were restimulated overnight at a ratio of 1:1 with irradiated PKH-2 (Sigma-Aldrich, Buchs, Switzerland)-labeled PHA blasts obtained by activation of non-irradiated stimulatory PBMCs with one µg/ml PHA (Gibco). As a control, part of the cells was also restimulated with autologous PHA blasts. The percentage of CD137-positive PKH-2 negative CD8-positive CD56-negative viable T cells was quantified by flow cytometry. The level of alloreactivity was measured as % CD137^+^CD8^+^ cells. It corresponds to the delta between the % CD137^+^CD8^+^ cells measured at day 14, after restimulation on day 13 with allogeneic cells, minus % CD137^+^CD8^+^ measured at day 14, after restimulation with autologous cells (14, 24). To upregulate the HLA expression of stimulator cells, stimulator cells were incubated in culture medium overnight with or without 50 ng/ml TNFα and 100 ng/ml IFNβ (PrepoTech, London, UK) prior irradiation and mixing with the responder cells.

### Immunofluorescence

To label activated cytotoxic CD8 cells, APC-labeled anti-human CD8a, (clone HT8a) PerCP/Cy5.5-labeled anti-human CD56 (clone HCD56) (BioLegend, Fell, Germany) and FITC-labeled anti-human CD137 (clone 4B4-1) (Milteny Biotec) antibodies, as well as APC- and FITC-labeled murine IgG1 isotype controls (clone MOPC) (BD Bioscience, Switzerland) were used.

HLA class I surface expression was determined on CD3^+^ T cells using the monoclonal antibodies APC-labeled anti-human CD3 and FITC-labeled anti-HLA-ABC (Miltenyi Biotec) and their corresponding isotype control. HLA- C surface expression was determined on CD3^+^ T cells using the monoclonal antibodies APC-labeled anti-human CD3 and anti-HLA-C (clone DT9) (Milliport, Darmstadt Germany) and FITC-labeled anti-mouse IgG2b and their corresponding isotype controls (Lucernachem, Luzern, Switzerland). Data acquisition was performed on gated mononuclear cells, using the ACCURI-C6 cytometer (BD) and the CFLOWPLUS analysis software (BD Bioscience, Allschwil, Switzerland).

### Cell Sorting

Activated CD8^+^ CD137^+^ T cells were sorted after staining with anti-CD137-FITC and anti-CD8-PEVio770 (clone BW135/80) (Miltenyi Biotec), as CD137-positive CD8-positive PKH negative cells on a BioRad s3 cell sorter (BioRad, Hercules, USA). The gating strategy is presented in **Supplementary Figure 1**. A mean of 6937 ± 5757 CD137+CD8 cells were isolated, depending on the strength of the alloresponse (average purity was 96.1 ± 1.5%).

### DNA Extraction of Sorted Cell

Genomic DNA was extracted using the Genomic DNA extraction kit NucleoSpin (Machery-Nagel, Düren, Germany)

### T-Cell Receptor Immunosequencing

High throughput sequencing of the TCR CDR3β region was carried out at survey resolution on the Illumina HiSeq system (Illumina, San Diego, USA) following a multiplex PCR (ImmunoSEQ^©^ assay, Adaptive Biotechnologies, Seattle, USA). We used 400 ng of DNA from unstimulated isolated CD8+ cells and the total amount of extracted DNA from sorted alloreactive CD137+CD8^+^ cells (see **Supplementary Table 1**). Sequencing results were sent to Adaptive Biotechnologies for analysis and datasets were downloaded from the Adaptive Biotechnologies platform for further investigations. Sample overview indicating number of productive templates, rearrangements, maximal productive frequencies and clonalities is provided in **Supplementary Table 1**. T-cell repertoire diversity was estimated by Shannon clonality, defining maximum diversity (i.e., polyclonal samples) at 0 and minimum diversity (i.e., monoclonal samples) at one. Analyses were performed using the online ImmunoSEQ Analyzer 3.0 software provided by Adaptive Biotechnologies. TCR overlap analyses were based on the amino acid sequences of the CDR3 region. Repertoire overlap between two samples (S1 and S2) was calculated with the following formulas: % TCR overlap is the number of shared clonotypes between S1 and S2 divided by the total number of clonotypes in S1 and S2. This formula is similar to the Jaccard index. Respective % overlap was calculated as the number of shared clonotypes between S1 and S2 divided by the number of clonotypes in S1 or S2, respectively. In addition, the Morisita’s index (25) was estimated with ImmunoSEQ Analyzer 3.0 software. This index measures the overlap based on the statistical dispersion of clonotypes in the samples and is expected to vary between 0 (no similarity) and 1 (complete similarity). To compare rearrangements (i.e., unique CDR3β amino acid sequences) with significantly increased or decreased frequencies between two samples or experimental conditions, binomial differential abundance analysis was performed with the ImmunoSEQ Analyzer 3.0 software and as specified in (26). Respective cumulative frequencies of shared clonotypes in different experiment conditions were calculated based on abundance scatter files. Scatterplots, barplots, and boxplots were generated using R version 3.5.1.

### Statistics

Paired *t*-tests were performed with GraphPad prism software version 8.01 (GraphPad, San Diego, CA, USA) A threshold of 5% was considered for statistical significance. Clonotypes with p-values lower than 0.01 were identified as being differentially abundant between two samples or experimental conditions according to the differential abundance tool.

## Results

To investigate alloreactive T-cell repertoires, peripheral blood mononuclear cells (PBMC) of HLA genotyped anonymous blood donors were cultured *in vitro* in a classical one-way MLR assay. After specific restimulation, CD8^+^ responder T cells expressing the activation antigen CD137 were isolated by flow cytometry and their repertoire determined by high throughput sequencing of the TCR CDR3β region.

### Clonotype Frequency Distribution of Alloreactive CD137+CD8+ T Cells

**Figure 1:**
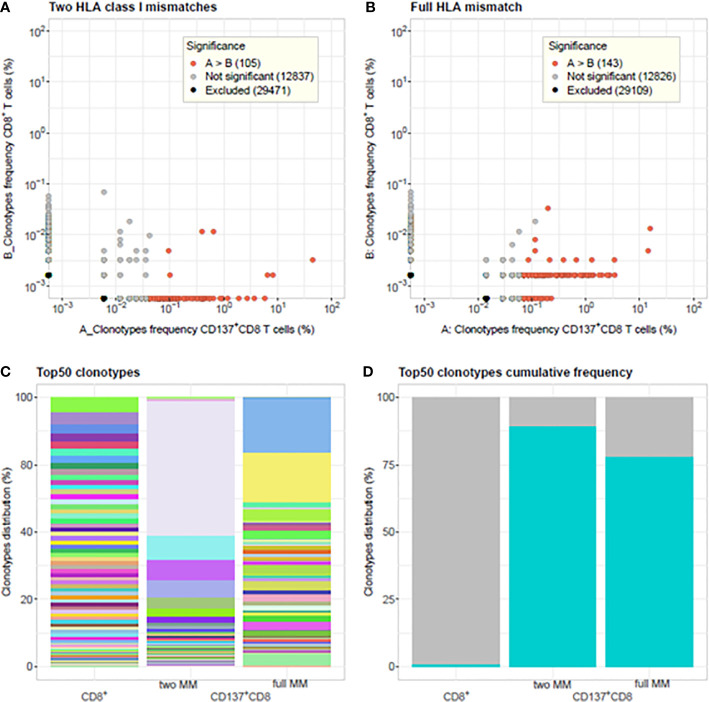
Comparison of T-cell clonotype frequency distributions between unstimulated CD8^+^ T cells and activated CD137^+^CD8^+^ T cells. Clonotype frequency scatterplots of CD8^+^ T cells isolated from unstimulated PBMC of the responder blood donor before culture and CD137^+^CD8^+^ T cells isolated at day 14 from MLR cultures between responder and stimulator cells harboring: **(A)** two HLA class I mismatches A*11:01/24:02 and C*01:01/04:01(i.e., 8/10 HLA matched, 2 MM) and **(B)** full HLA mismatch (i.e., 0/10 HLA matched, full MM). In **(A)** 4.68% and in **(B)** 31.97% of the CD137^+^CD8^+^ clonotypes are shared with the unstimulated CD8^+^ T-cells clonotypes. The cumulative frequencies of the shared CD137^+^CD8^+^ clonotypes observed in the unstimulated population are in **(A)** 0.61 and in **(B)** 0.88. The differential abundance tool of ImmunoSEQ Analyzer 3.0 was used to analyze clonotype frequencies (26). Red dots represent clonotypes that are observed with a statistically significant greater frequency in sample A compared to sample **(B)** Grey dots represent clonotypes that are not found to be differentially abundant. Black dots represent clonotypes that are excluded from the analyses. **(C)** Barplots showing the % clonal frequency distribution of the 50 most frequent clonotypes in unstimulated CD8^+^ T cells versus CD137^+^CD8^+^ activated cells of MLR with two HLA class I mismatches and full HLA mismatch. Same colors represent same clonotypes. **(D)** Cumulative frequency of the top 50 rearrangements (cyan-blue bars) from unstimulated CD8^+^ T cells (1.2%), CD137^+^CD8^+^ activated cells in 2 HLA class I MM MLR (89.5%) and full MM MLR (78.4%), respectively. TCR clonalities are 0.006, 0.55, and 0.31 for the unstimulated CD8^+^ T cells, the CD137^+^CD8^+^ T cells in the 2 HLA class I MM, and full MM MLR, respectively. Morisita indices among the three samples are <0.0015.

**Figure 2:**
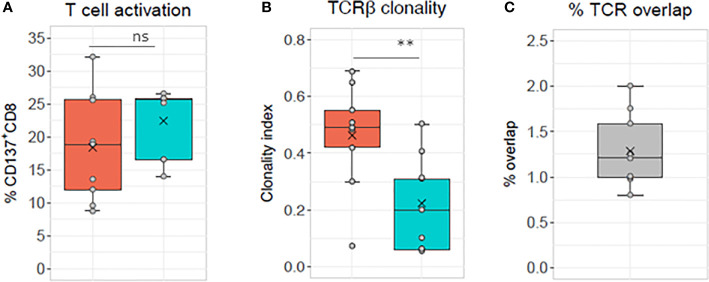
TCR analyses of sorted CD137^+^CD8^+^ T cells. The same responder cells are stimulated with allogeneic cells mismatched for either one or two HLA alleles (orange box, i.e., 8–9/10 HLA matched, 1–2 MM) or for all HLA alleles (blue box, i.e., 0/10 HLA matched, full MM) in nine different MLR cultures (see **Supplementary Table 2** for HLA genotyping of the cells used for the experiments and **Supplementary Table 1** for TCR sequencing sample overview). **(A)** Percentage of CD137^+^CD8^+^ cells measured after specific minus autologous restimulation. Mean ± SD: 1–2 MM 18.39 ± 8.1, full MM 22.43 ± 5.1, ns, not significant paired *t* test p>0.05. **(B)** TCR clonality mean ± SD: 1–2 MM 0.46 ± 0.18, full MM 0.22 ± 0.16, **significant paired *t* test p = 0.01 and **(C)** percentage of CD137^+^CD8^+^ T cell clonotypes shared between the two culture conditions (mean, 1.28 ± 0.4).

Alloreactive repertoires were determined in nine different MLR cultures and compared between responder cells, which were either activated with allogeneic stimulator cells harboring one or two HLA class I mismatches or with fully HLA mismatched stimulator cells (**Figures 1** and **2** and **Supplementary Tables 1** and **2**). In **Figures 1A, B**, representative of two prototypical MLR cultures, we observe the presence of a majority of low frequency (<0.1%) clonotypes in the unstimulated CD8^+^ population of the responder isolated before the MLR cultures. By contrast, after allogeneic stimulation, most clonotypes were retrieved at much higher frequencies (up to 74.6%) in the alloreactive CD137^+^CD8^+^ responder population. Furthermore, some clonotypes with specific TCR rearrangements, although not detected in the unstimulated CD8^+^ population, were isolated after stimulation representing the expansion of very low-frequency (< 0.001%) clonotypes. Conversely, some other clonotypes were observed only in the unstimulated population but not after stimulation. Overall, repertoire overlap between unstimulated and activated cells was very low, with Morisita indices below 0.0015. Compared to unstimulated cells, activated CD137^+^CD8^+^ T-cell repertoires showed a substantial increase in clonality: from 0.006 to 0.55 (**Figure 1A**) and from 0.006 to 0.31 (**Figure 1B**), respectively. The clonal distribution of unstimulated cells was even with clonotype frequencies varying between 0.001% and 0.1%, while a few dominant clonotypes were observed at much higher frequencies in activated cells (**Figure 1C**). The cumulative frequencies of the clonotypes retrieved after allogeneic stimulation and observed in the unstimulated population are of 61% and 88% in the two MLR, respectively. This is characteristic of the strong alloreactive expansion to high frequencies of a few clonotypes among a broad repertoire of low frequency clonotypes in the unstimulated T-cell population. Accordingly, while the 50 most frequent clonotypes in unstimulated CD8 cells represent 1.2% of all the clonotypes, they represent more than 78% of the clonotypes retrieved from activated CD137^+^CD8^+^ cells (**Figure 1D**). The proportion of specifically activated cells was not significantly (p>0.05) different whether the responder cells were stimulated with cells harboring one or two (mean, 18.39 ± 8.1) HLA mismatched alleles or cells fully (mean, 22.43 ± 5.1) HLA mismatched (**Figure 2A**). By contrast, the clonality was significantly lower in cells activated with fully mismatched stimulator cells (mean, 0.22 ± 0.16) than stimulator cells with one or two HLA class I mismatches (mean, 0.46 ± 0.18, p = 0.01, **Figure 2B**). Note: the CDR3β amino acid identity, referred to in this study as “% TCR overlap” (also named Jaccard index), represents the percentage of clonotypes shared among the total number of clonotypes observed in two repertoires under comparison. **Figure 2C** shows that the % TCR overlap between the activated CD137^+^CD8^+^ T cells in one to two HLA class I mismatched or fully mismatched MLR was low in all MLR pairs (mean, 1.28 ± 0.4%).

We investigated in a few MLR cultures whether CMV-specific T cell clonotypes could be enriched after the *in vitro* stimulation. However, based on a set of 18,855 potentially public CMV-specific T-cell clonotypes gathered from two resources (27, 28), we did not observed over representation of such clonotypes (data not shown).

### Reproducibility of Clonal Stimulation

To evaluate whether the low TCR overlap observed in different MLR conditions is genuinely a consistent result, we monitored our experiments’ reproducibility. Interestingly, MLR repeats showed heterogeneous CDR3β clonal distributions, although similar ranges of T-cell activation and clonality were retrieved (**Supplementary Table 1A** and **3** and **Supplementary Figure 2**). Nevertheless, up to 10% of shared CD137+CD8+ T-cell clonotypes were detected in triplicate MLRs. In pairwise comparisons, TCR overlap as high as 23.6% was observed, also reflected by Morisita’s index up to 0.45 and respective cumulative frequencies of shared clonotypes up to 83% and above 50% in all but one replicate. Shared and non-shared clonotypes among the replicates were present across all ranges of frequencies.

### T-Cell Receptor Repertoires of Alloreactive T-cell Clonotypes in Mixed Lymphocyte Reaction Cultures

**Figure 3:**
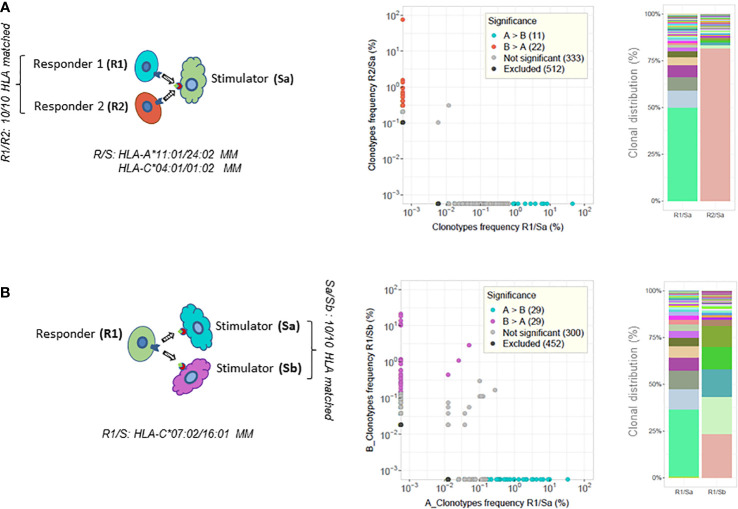
TCR analysis of paired MLRs. CD137^+^CD8^+^ T-cell clonotype frequency scatterplot comparisons between paired MLRs and clonal distribution of the top 50 most frequent clonotypes retrieved from each culture (illustrative examples of results taken from **Table 1**). **(A)** Two MLR cultures of different HLA 10/10 matched responder cells (R1 or R2) stimulated by the same mismatched stimulator cells (Sa). R1/R2 cells are HLA-A*11:01 versus 24:02 and HLA-C*04:01 versus 01:02 mismatched with Sa cells. **(B)** Two MLR cultures of the same responder cells (R1) stimulated with cells from two distinct stimulators (Sa and Sb). Sa and Sb are 10/10 HLA matched. R1 cells are HLA-C*07:02 versus 16:01 mismatched with Sa/Sb cells. The top 50 clonotypes represent 89.5% in R1/Sa and 82.3% in R2/Sa (top experiments shown in panel **A**), and 91.9% in R1/Sa and 90.5% in R1/Sb (bottom experiments shown in panel **B**) of the respective repertoires. Colored dots in scatterplots represent clonotypes with frequencies differing significantly between paired MLRs. Cyan-blue dots represent clonotypes observed with a statistically significant greater frequency in sample A compared to sample B, red and purple dots clonotypes have a statistically significant greater frequency in sample B compared to sample A, while gray dots represent clonotypes not differing significantly in frequency. Black dots clonotypes are excluded from the analyses. Frequency analysis was performed with the differential abundance tool in ImmunoSEQAnalyzer 3.0. Barplots represent the clonal distribution of the 50 most frequent clonotypes.

To further investigate the allogeneic repertoire specificity, different combinations of HLA mismatched responder/stimulator cells were tested in distinct MLR cultures. The TCR repertoires of purified activated CD137^+^CD8^+^ cells were sequenced and analyzed (**Figure 3** and **Table 1**).

**Table 1:** Paired alloreactive T-cell response.

MLR^a)^		HLA MM^b)^	% CD137^+^CD8	TCR clonality	%TCR overlap^c)^	Morisita index	Respective% TCR overlap^d)^	Cumulative freq. of shared clonotypes
Responder 1	Stimulator a	HLA-A*11:01 vs 24:02 HLA-C*04:01 vs 01:02	12.0	0.55	0.2	10^−6^	0.3	0.0002
Responder 2		HLA-A*11:01 vs 24:02 HLA-C*04:01 vs 01:02	8.8	0.64			1.6	0.004
Responder 3	Stimulator b	HLA-C*07:01 vs 12:03	12.9	0.31	0	–	–	–
Responder 4		HLA-C*07:01 vs 12:03	13.1	0.30			–	–
Responder 5^e)^	Stimulator c	HLA-C*07:02 vs 16:01	25.6	0.51	1.9	0.0002	3.1	0.0099
	Stimulator d	HLA-C*07:02 vs 16:01	18.8	0.49			4.6	0.054
Responder 5^e)^	Stimulator c	HLA-C*07:02 vs 16:01	22.2	0.38	1.9	0.044	4.6	0.10
	Stimulator d	HLA-C*07:02 vs 16:01	11.3	0.41			3.1	0.08
Responder 3	Stimulator b	HLA-C*07:01 vs 12:03	12.9	0.31	1.7	0.022	5.8	0.10
	Stimulator e	HLA-C*07:01 vs 12:03	13.5	0.31			2.3	0.05

^a)^Responder 1 cells are 10/10 HLA matched with responder 2 cells, dito responder 3 with responder 4, stimulator c with stimulator d and stimulator b with stimulator e cells.

^b)^mismatched HLA class I alleles between responder and stimulator cells.

^c)^total % shared T cell clonotypes.

^d)^% shared T cell clonotypes in one MLR compared to the other MLR.

^e)^MLRs performed in duplicate.

Responder 1and responder 2 TCR clonalities of unstimulated CD8+ T cells are 0.006 and 0.023 respectively with 0.1% overlapping T-cell clonotypes and responder 3 and responder 4 TCR clonalities are 0.04 and 0.06, respectively, with 1,1% overlapping T-cell clonotypes. TCR clonality of responder 5 cells is 0.026.

First, responder T cells isolated from two distinct HLA identical blood donors (i.e., HLA-A, B, C, DRB1, DRB3/4/5, and DQB1 matched at high resolution) were stimulated in parallel cultures by cells isolated from a third HLA mismatched donor (see the illustrative chart in **Figure 3A** and the top half of **Table 1**). Second, cells isolated from one donor were stimulated in parallel cultures with HLA mismatched stimulator cells isolated from two HLA identical donors (see the illustrative chart in **Figure 3B** and bottom part of **Table 1**).

As shown in **Table 1**, the clonality and percentage of activated CD137^+^CD8^+^ T cells were constrained within close ranges of values for each pair of MLRs. Interestingly, the percentage of activated clonotypes sharing a CDR3β amino acid sequence (i.e., % TCR overlap) was low when comparing pairs of MLR culture performed at the same time in parallel, ranging between 0 and 1.9%. In addition, the percentage of activated clonotypes shared in one MLR culture compared to the other culture ranged between 0 and 5.8% (i.e., respective % TCR overlap). Along this line, very low cumulative frequencies of shared clonotypes were measured between pairs of MLR. Accordingly, the Morisita’s indices were very low and varied from 10^−6^ to 0.044. The clonal distribution of the 50 most frequent clonotypes revealed dominant clonotypes in each MLR condition (**Figure 3**, right panel), but these clonotypes did not share the same CDR3β amino acid sequence. Of note, the CD8^+^ T-cell repertoires of the two HLA matched responders’ pairs (responders 1 and 2 or responders 3 and 4, respectively) were very distinct sharing only 0.1% to 1.1% of TCR rearrangements (results not shown).

### Effect of Human Leukocyte Antigen Upregulation on the T-Cell Receptor Repertoire of the Alloreactive T-cell Response

Under *in vivo* conditions, HLA expression, which has previously been shown to influence the allogeneic immune response (14–16), might be affected by inflammation driven by infection or GVHD. The HLA expression of stimulator cells was upregulated *in vitro* by overnight incubation with TNFα alpha and IFNβ to mimic inflammation. RNA sequencing (results not shown) and cytofluorometric analysis (**Supplementary Figure 3**) revealed that, although expressed at variable levels, all HLA class I alleles, including HLA-C alleles, were upregulated after TNFα/IFNβ induction to similar extent. This was confirmed by correlation coefficients of r = 0.84 and r = 0.92 between unstimulated and upregulated HLA-ABC and HLA-C expression, respectively. The mean fold cell surface expression upregulation measured on PBMCs, isolated from 56 different blood donors, was 1.62 for total HLA class antigens and 1.9 for HLA-C antigens on PBMCs from 32 of these 56 blood donors. Compared to untreated stimulator cells, cytokine TNFα/IFNβ treated stimulator cells, induced similar percentages of activated CD137^+^CD8^+^ T cells in 20 parallel MLR cultures (**Supplementary Figure 4**). The mean percentage of CD137^+^CD8^+^ T cells were 18.5 ± 9.8 and 14.6 ± 11.2 (p = 0.051) in MLR cultures of untreated versus TNFα/IFNβ treated stimulator cells, although the cell surface expression was significantly increased in treated stimulator cells (p<0.0001).

**Figure 4:**
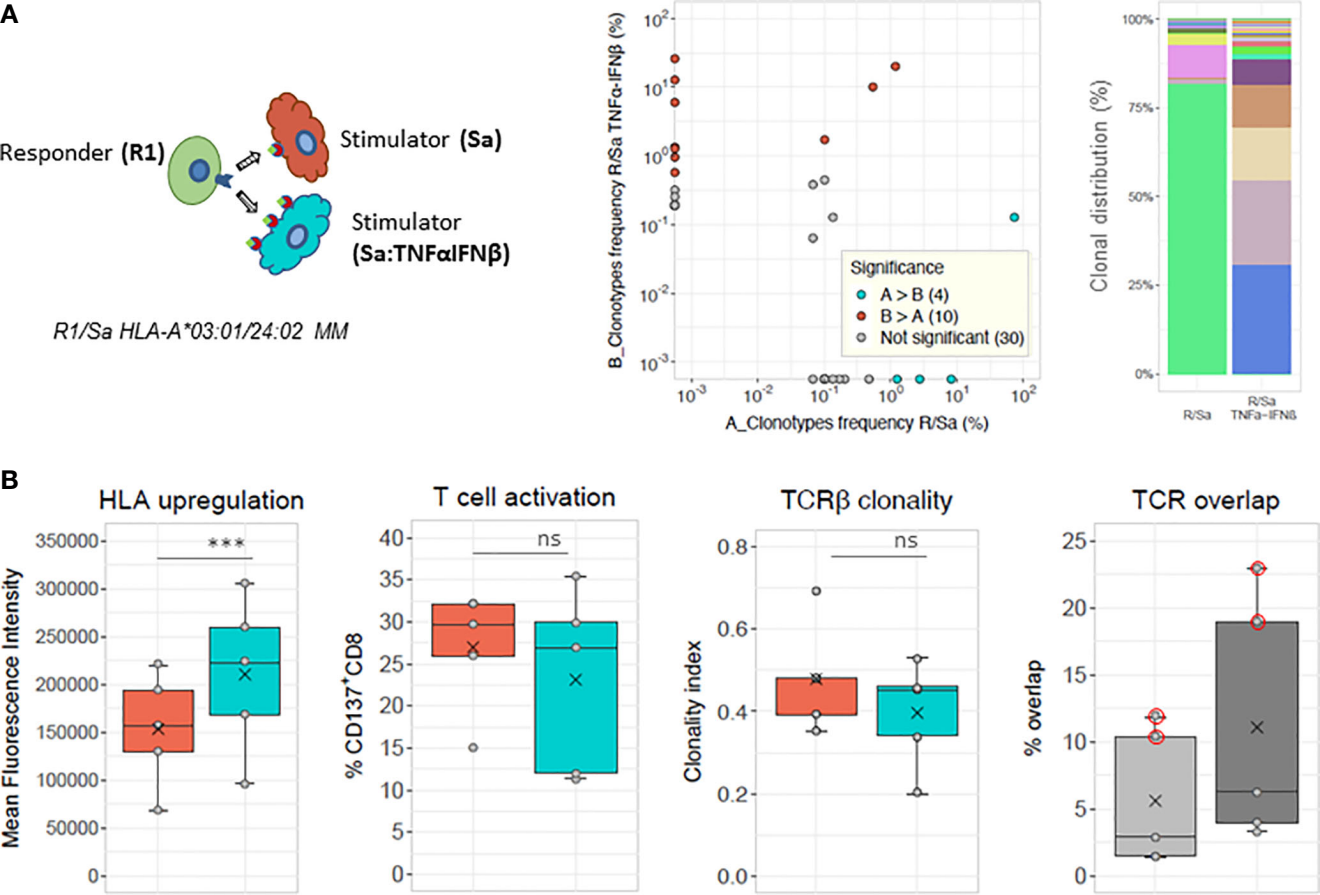
Modulation of allogeneic T cell response by HLA class I upregulation. **(A)** Chart of MLR stimulations: responder cells (R) derived from the same blood donor are stimulated in parallel cultures with allogeneic stimulator cells, with basal (Sa) or upregulated HLA surface expression after overnight TNFα and IFNβ cytokine treatment (Sa: TNFαINFβ). Scatterplot and barplot are a representative example of one of the five parallel MLRs shown in **(B)**. Frequency scatterplot showing 1.5% (8/531) of shared clonotypes between the two conditions. Colored dots in scatterplots represent clonotypes with frequencies differing significantly between paired MLRs (i.e., cyan-blue dots represent clonotypes that have a statistically significant greater frequency in sample A compared to sample B, red dots clonotypes have a statistically significant greater frequency in sample B compared to sample A), while gray dots represent clonotypes not differing significantly in frequency. The frequency analysis was performed with the differential abundance tool in ImmunoSEQAnalyzer 3.0). The cumulative frequency of the shared clonotypes in MLR stimulation with Sa : TNFαINFβ is 0.33 and with untreated Sa is 0.75, respectively. (Right panel) Clonal distribution of the top 50 most frequent clonotypes in each culture (i.e., with basal (R/Sa) or upregulated (R/SaTNFαINFβ) HLA cell surface expression of stimulator cells). Responder and stimulator cells are mismatched for HLA-A*03:01 versus 24:02. The top 50 clonotypes represent: 91.3% in R/Sa and 87.4% in R/Sa+TNFαINFβ of the respective repertoires. **(B)** HLA cell surface upregulation, T-cell activation, TCRβ clonality and % TCR overlap determined in five pairs of MLRs performed with stimulator cells treated with TNFα and IFNβ (cyan-blue boxes) or not treated before stimulation (orange boxes). The light and dark gray boxes in the last panel represent total and respective TCR overlap (in %), respectively. The mean value of Morisita’s indices for TCR overlap is 0.024. Of note, among the five pairs of MLR, two pairs were performed with fully HLA mismatched stimulator cells, while the other stimulator cells were mismatched for one HLA-C (07:02 versus 16:01) or one HLA-A (03:01 versus 24:02) allele. TCR overlap was determined based on CD137^+^CD8^+^ T cells except in two out of the five MLR cultures where total CD8^+^ T cells were isolated. This is shown by the red circled dots on the lower right panel. No statistical differences (ns) in T-cell activation or TCR clonality is observed between MLRs with basal or upregulated HLA expression (paired *t*-tests). ***^)^ HLA induced upregulation was statistically significant (paired *t* test, p = 0.0053).

The clonal analysis of alloreactive CD137^+^CD8^+^ T cells induced by the same mismatched HLA stimulator cells with basal or elevated HLA antigen expression is shown for one representative experiment in the upper panels of **Figure 4**. The percentage of shared clonotypes was only 1.5% (Morisita index of 0.011). Cumulative frequencies of clonotypes shared was of 33% in the MLR culture toward cells with upregulated HLA expression compared with clonotypes of the paired MLR toward cells of basal HLA class I expression. Of note, four shared clonotypes were present at frequencies above 0.01 in one or the other cultures. The clonal distribution of the 50 most frequent clonotypes is represented in **Figure 4** (upper right panel) and shows a few unshared dominant clonotypes in both culture conditions. The results of five different MLR cultures with basal or elevated HLA antigen expression are shown in **Figure 4** (lower panels). The mean percentage and clonality of activated CD137^+^CD8^+^ T cells did not significantly differ when induced by stimulating cells treated or not with the cytokines. The % TCR overlap of activated CD37^+^CD8^+^ cells in untreated and treated conditions was low and ranged between 1.4% and 2.9% in three out of the five cultures. However, a slightly higher proportion (3.3% to 6.3%) of the TCR rearrangements was observed in the CD137^+^CD8^+^ T cells induced by the TNFα/IFNβ treated stimulator compared to the cells detected in the parallel MLR induced by untreated stimulator cells (**Figure 4**, lower, right panel). The cumulative frequencies of the shared clonotypes between cultures stimulated with TNFα/IFNβ treated stimulator versus untreated stimulator cells was 0.18 ± 0.15. It is important to note that in two out of the five cultures (**Figure 4**, dots circled in red), the repertoires were not analyzed based on activated CD137^+^CD8^+^ cells but on the total of CD8^+^ sorted responder T cells, as the number of CD137^+^CD8^+^ cells was to low to be sorted. This explains the greater overlap ranging between 10.4% and 22.9% in these two experiments.

## Discussion

The data of this study suggest that in donor/recipient HLA mismatched situations (like it might occur in HSCT), the same mismatched stimulatory cells (in the case of HSCT: recipient antigen-presenting cells) induce the proliferation of different responder T-cell clonotypes (donor T-cell clonotypes) with a little percentage of shared TCRs in distinct HLA identical (i.e., 10/10 matched) responder cells. Similarly, distinct HLA identical (i.e., 10/10 matched) stimulators cells (recipient antigen-presenting cells) induce the proliferation of different responders TCR clonotypes (donor T-cell clonotypes) and repertoire in the same HLA mismatched responder/stimulator configuration. Random selection and expansion of alloreactive T-cell clonotypes were also observed in MLR repeats and triplicates, although at a lesser extent Moreover, the level of mismatched HLA cytokine-modulated expression on the cell surface does not influence the strength of the T-cell response, but it affects the repertoire of the alloreactive CD137^+^CD8^+^ T cells retrieved after culture. Our results are in line with previous reports (3–5, 17) indicating the very high flexibility of alloreactive TCRs in the alloimmune response, as well as the random selection and expansion of alloreactive cytotoxic T-cell clones, which is influenced by the complex interplay between the TCRs and mismatched-HLA-peptide complexes, alongside additional factors such as the fitness of clones, cytokines and inflammatory events.

To monitor the cytotoxic T-cell response in transplantation, T cells were isolated from defined HLA genotyped healthy donors and stimulated with specific HLA-mismatched allogeneic cells *in vitro*. We are aware that this study’s *in vitro* model might represent only a snapshot of the possible *in vivo* alloimmune response. Indeed, the observed expansion of dominant clonotypes can be affected by the clones’ microenvironment and fitness. In this study, the starting PBMC number was 2 million and might not always include the total number of cells with alloreactive potential. This is especially true when considering that the clonotypes observed with increased frequencies in the responding cell population were not necessarily detected in the starting population because of their supposed too low frequencies. Only an increased number of cultures performed in parallel would probably clarify this matter and minimize technical limitations (29–31). However, working with a higher number of cells from two individuals with a single HLA mismatch would be cumbersome and require higher blood withdrawals, which is not ethically acceptable. Emerson et al. (32) distinguished low (i.e., not seen before stimulation and representing 84% of the reacting clonotypes) and high abundance clonotypes (i.e., seen before and after stimulation) in the alloresponse of the total T-cell population. They reported repetitive detection of the high abundance clonotypes only.

Similarly to others (33), we observed shared and non-shared clonotypes at various frequencies in activated CD137^+^CD8^+^ cells of MLR triplicates (**Supplementary Figure 2**). Conscious of the technical restrictions, we expected that the very abundant alloreactive clones would be repeatedly detected, and we have reduced the possible randomization of the results by testing multiple alloresponses toward different single HLA antigens. With these precautions in mind, we are confident that the results obtained in this study stand to estimate events occurring during the alloimmune response. To avoid inter-individual variability and to better define the diversity of the TCR repertoire of alloreactive cells, distinct stimulations of the same cells were examined. Furthermore, alloreactive activated CD137^+^CD8^+^ T cells were sorted after being specifically restimulated to minimize bystander stimulation of non-specific clones. We can however not exclude that, while sorting activated cells, we also sorted some auto-specific cells. Note that when measuring T cell activation, we did stimulate the cultures with autologous cells to monitor specificity. Accordingly, the referred activation represents % CD137^+^CD8^+^ in specifically restimulated cultures minus % CD137^+^CD8^+^ T cells in the same culture after autologous restimulation. The % after autologous stimulation never exceeded 10% of CD137^+^CD8^+^ (data not shown).

Using such assays and similar to others (21, 22, 33), we identified highly frequent clonotypes among alloreactive CD8^+^ T cells previously not detected in unstimulated cells. This confirms the highly efficient expansion of reactive T-cell clones in the allogeneic immune response. The increased clonality observed in **Figures 1** and **2** for T cells stimulated with a single or a few alloantigens compared to T cells stimulated with a large number of alloantigens is concordant with the results of de Wolf et al. (21). These authors reported a reduced diversity and thus increased clonality of the alloresponsive CD4^+^ and CD8^+^ T cells stimulated by haploidentical cells (i.e., half-matched for HLA) as compared to cells stimulated with fully HLA mismatched cells. This supports the belief that a higher number of HLA mismatches correspond to a higher number of potential alloantigens, thus inducing a more diverse alloreactive repertoire at fault for a stronger immune response in transplantation. Nevertheless, it has to be kept in mind that a single mismatched HLA molecule might bind a large variety of peptides and may represent many different alloantigens, possibly interacting with different TCRs. On the other side of this multifaceted ligand/receptor cognate interaction, one given TCR was reported to react with more than 100 different peptides (17, 34). Other authors reported up to 1 million peptides being potentially recognized by each TCR. This TCR’s particularity probably also influences the specificity of the alloresponse. In an HLA class II mismatched situation, Arrieta-Bolanos et al. (22) reported that although higher levels of alloresponse were detected in the same HLA-DPB1*04:02 individual after stimulation with Hela cells expressing HLA-DPB1*09:01 rather than HLA-DPB1*02:01, very different clonotypes of similar TCR clonality were measured among the responding CD4+ T cells. Their results suggest that the number of amino acid mismatches between the HLA of the responder and stimulator cell does not influence the clonality of the responding T cells, leading to the same conclusion as we do regarding the pleiotropy of the TCR repertoire when stimulated under similar controlled conditions.

Furthermore, in this study, we have described that the very same HLA mismatched stimulatory cells (thus expressing the same HLA-peptide alloantigens) induce the proliferation of clonotypes with a very low percentage of shared TCR in distinct but HLA 10/10 matched responder cells. This was also the case when the alloresponse was restricted to a single allelic mismatch. These results suggest an individual selection of these clonotypes, which could be influenced by environmental factors and/or immunological memory of the responding cells’ past immune responses, similar to the phenomenon described in twins (35). The preferential thymic selection of different repertoires as well as the distinctive memory versus naïve CD8^+^ T-cell repertoires could also influence the results (36, 37). Likewise to us, Arrieta-Bolanos et al. (22) reported that the same HLA-DP mismatched expressing cells induce CD4^+^ T cells with different TCR rearrangements in different individuals.

We have also shown that HLA identical (10/10 matched) stimulatory cells isolated from distinct healthy donors induced the proliferation of different T-cell clonotypes in the same HLA mismatched responder cells. Such diversity stands for the “peptide-centric model” described by Cole and al (38). as it probably reflects the T-cell response toward heterogeneous peptidomes bound by the same HLA alleles of different individuals. A refined peptidome analysis would be required to investigate this hypothesis further. Along this line, Koyama et al. (39) reported that different T-cell clonotypes were detected in patients undergoing GVHD in the skin, colon, or blood of the very same patient, suggesting that HLA molecules present distinct peptides in various organs. In contrast, Michalek et al. (40) reported the expansion of a unique specific CD4^+^ T-cell clone in the blood of an HSCT patient undergoing GVHD.

Since additional factors such as concomitant infection or any ongoing inflammatory processes could play a role in the *in vivo* allogeneic response, we have monitored inflammatory cytokines’ effect on the alloresponse. To do so, the levels of HLA expression of stimulatory cells were boosted *in vitro* with a combination of TNFα and IFNβ. We could observe that all HLA class I alleles’ transcription (results not shown) and translation were increased with the same order of magnitude. Whether increased HLA expression alters the peptidome presented, has not yet been investigated. Nevertheless, comparing the alloresponses of the same CD8^+^ T cells induced by stimulating cells with or without upregulated HLA surface expression revealed no significant change in the response’s strength and clonality. Some TCR rearrangements were shared, revealing mean cumulative frequencies of 0.38 ± 0.32 and 0.18 ± 0.15 for shared clonotypes of the same CD8+ T cells induced by stimulating cells without or with upregulated HLA surface expression. The overlap of activated CD8^+^ cells in these two conditions was however weak, with a maximum of 6.3% of shared clonotypes. Although the low overlap might be influenced by the randomness observed in culture repeats, it supports a random selection and expansion of responding T cells with high TCR flexibility to bind foreign peptide-HLA complexes in alloimmune processes. Additionally, the results suggest that inflammation, occurring for example, after a bystander viral infection in transplantation, might also alter the allogeneic immune response as it might induce the activation of new responding alloreactive T cells. Potential effect of allele-specific expression which is a topic of debate (41–43) has not been investigated in this study.

Our study focused on specific HLA class I incompatibilities, mainly at the HLA-C locus. This could be considered a limitation of the study, however as HLA mismatched transplantations at the HLA-C locus have been privileged for many years in our institution, while HLA-B mismatches avoided, we did not have cells to perform HLA-B mismatch experiments and only a few experiments could involve HLA-A incompatibilities. In addition, it was not possible to assess every HLA-C mismatch combination due to the extensive HLA polymorphism. In HSCT, it is well known that particular mismatch combinations are so-called “permissive” and are leading to different transplantation outcomes (10, 14, 44). In our previous study (14), permissive HLA mismatched combinations did not induce CD137^+^CD8^+^ positive T cells suggesting any TCR repertoire bias before or after stimulation.

In conclusion, our data demonstrated the vast diversity of the alloimmune response regarding the expansion of T-cell clones. However, we are aware of the limitation of *in vitro* studies not allowing to fully extrapolate to *in vivo* situations. Factors that influence this expansion such as infection, immunosuppression and GVHD are under investigation (45–47). Interestingly, we recently presumed about such factors when we reported that the expansion of TCR clones in reconstructing the repertoire after HSCT was correlated to CMV reactivation in patients one-year post-HSCT, without being CMV specific (45). Another recent publication also demonstrates the difficulties to clearly associate the TCR repertoire and clinical events (46). Thus, the prediction of alloreactive T-cell response based on the TCR repertoire before and after transplantation remains a major challenge in HLA mismatched situations. Clinical protocols or pharmacological agents targeting specifically alloreactive T cells to control clinical complications such as GVHD or the graft-versus-leukemia effect (GVL) could be very challenging to establish.

## Data Availability Statement

The data sets presented in this study can be found in online repositories. The names of the repository/repositories and accession number(s) can be found in the article/**Supplementary Material**. DOI: 10.21417/FSB2020FI. The URL is: clients.adaptivebiotech.com/pub/ studer-bettens-2020-fi.

## Ethics Statement

The studies involving human participants were reviewed and approved by Ethical committee of University Hospitals of Geneva (CER 06–208 and 08–208R). Written informed consent for participation was not required for this study in accordance with the national legislation and the institutional requirements.

## Author Contributions

FB designed research studies, conducted experiments, and acquired and analyzed the data. ZS made the graphs. FB, SB, and JV wrote the manuscript. All authors contributed to the article and approved the submitted version.

## Funding

This study was supported by the Swiss National Science Foundation (grant #310030_173237/1), the Fondation privée of HUG, IRGHET (International Research Group on unrelated Hematopoietic stem cell Transplantation), and the Dr. Henri Dubois-Ferrière Dinu Lippatti foundation.

## Conflict of Interest

The authors declare that the research was conducted in the absence of any commercial or financial relationships that could be construed as a potential conflict of interest.
