# Supplementary material for: CD8+ T-Cell Repertoire in Human Leukocyte Antigen Class I-Mismatched Alloreactive Immune Response
Source: Front Immunol. 2021 Jan 20;11:588741. doi: 10.3389/fimmu.2020.588741 (PMC7856301; doi:10.3389/fimmu.2020.588741)
Supplement: Supplementary file 1 [file DataSheet_1.docx]

Supplementary Table 1A **Sample Overview**

Supplementary Table 1B **Sample Overview**

Supplementary Table 2 **HLA genotyping of PBMC of Figure 2**

Supplementary Table 3 **MLR replicates**


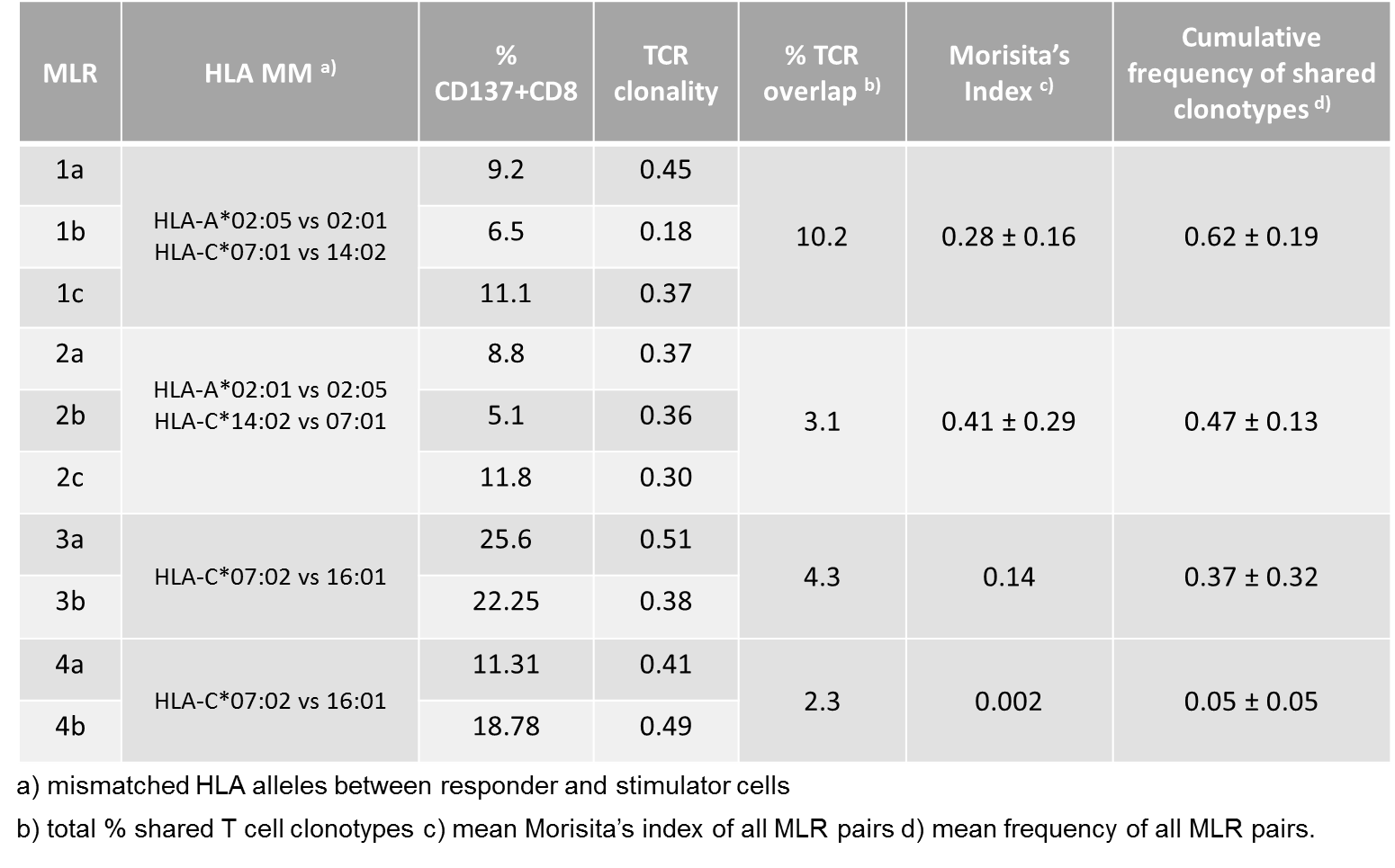


Supplementary Figure 1 **FACS gating strategy for sorting PKH^-^CD137^+^CD8^+^ cells**


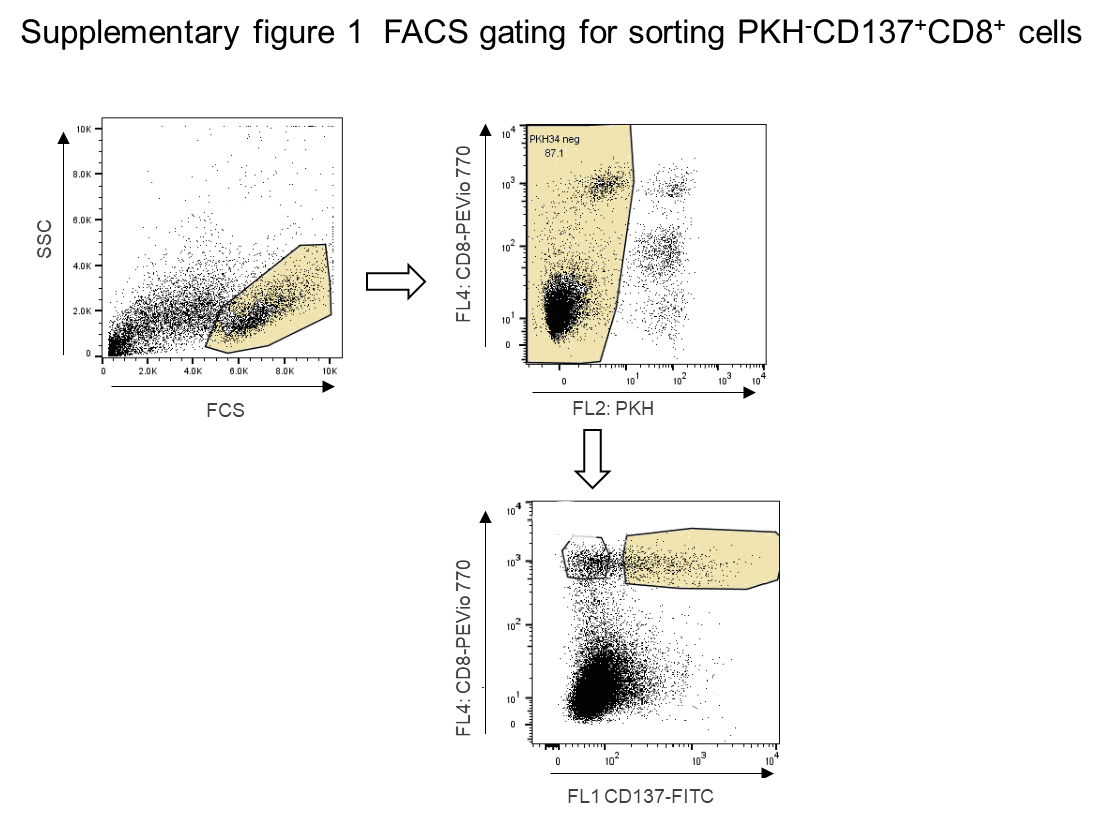


Supplementary Figure 2 **MLR culture repeats**


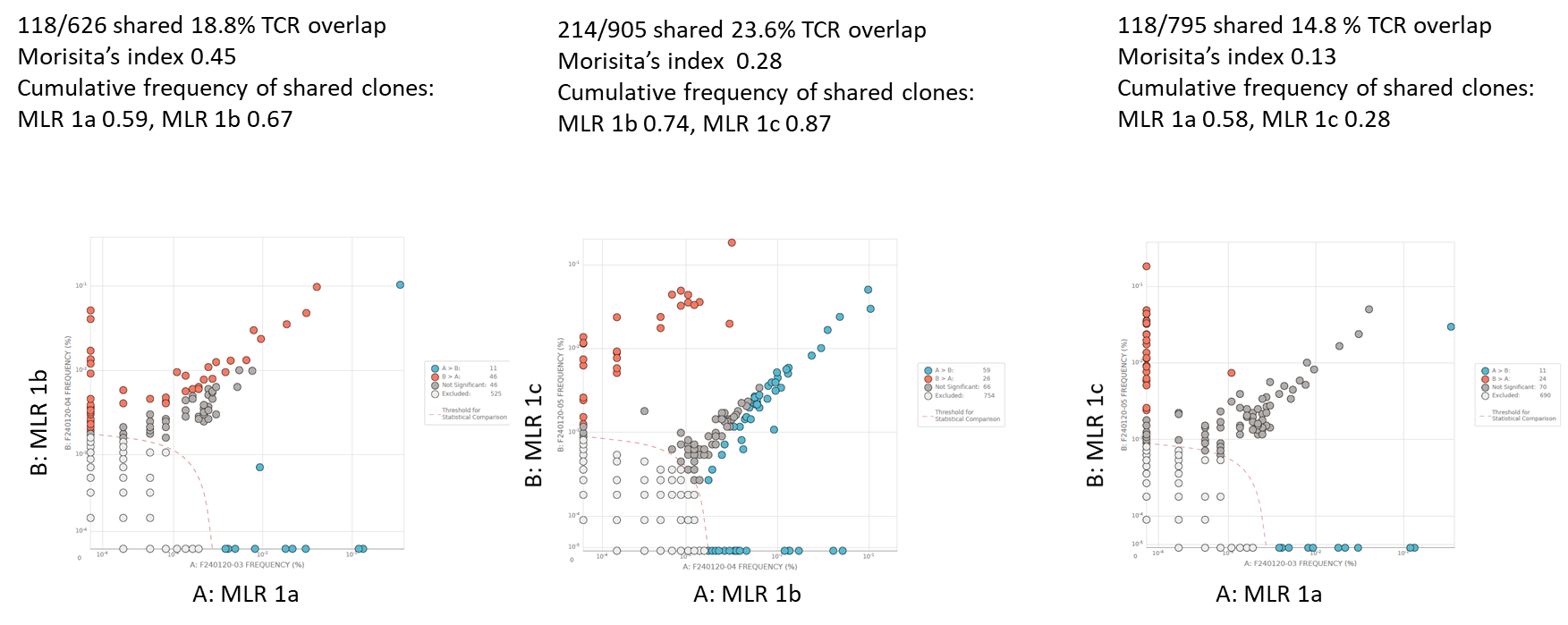


Supplementary Figure 3 **TNFα/IFNβ induced HLA class I upregulation**


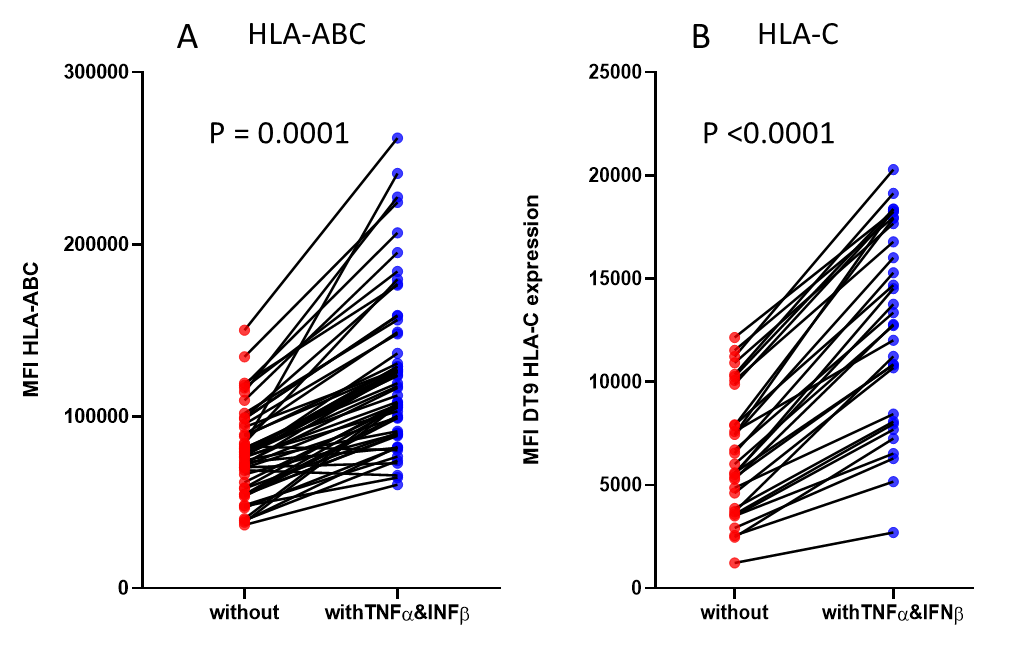

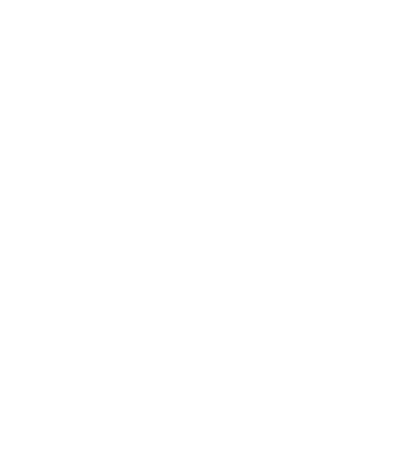


Supplementary Figure 4 **TNFα/IFNβ effect on alloreactive immune response**


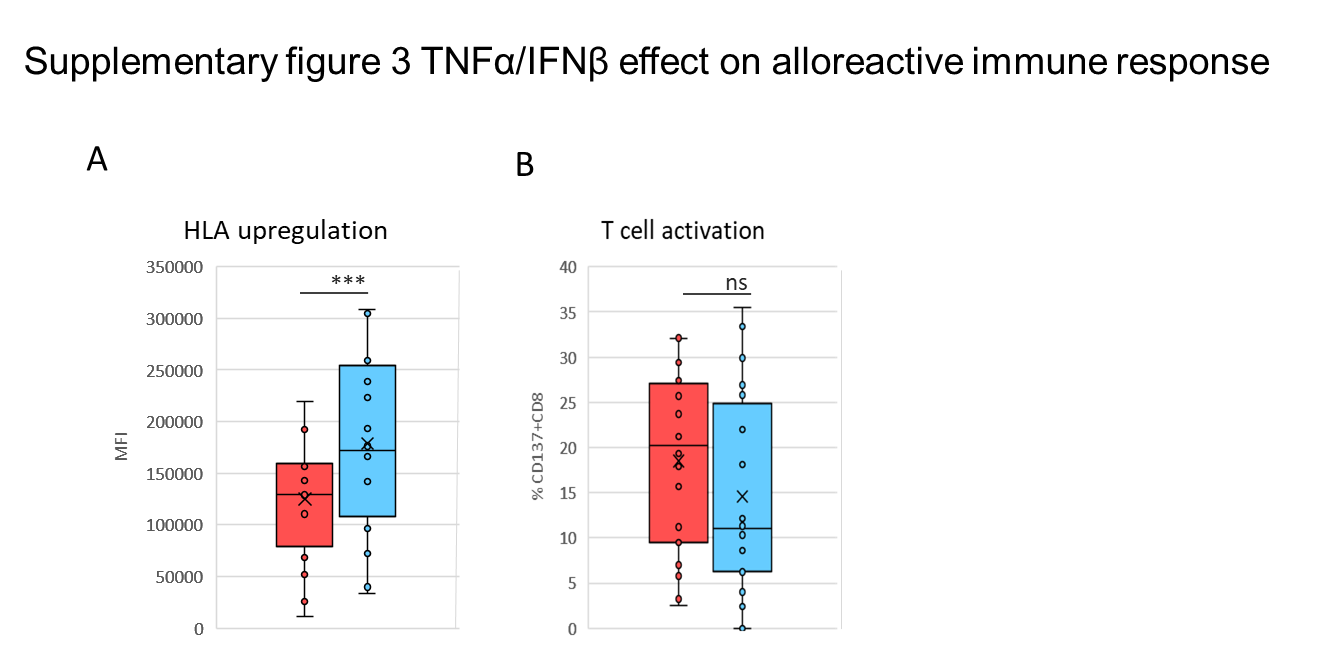


**Legends of Supplementary Figures**

Supplementary Figure 1: **Gating strategy** for sorting alloreactive CD137^+^CD8^+^ T cells. Cells are sorted on a Biorad S3 cell sorter according to the yellow gates. Mean cell purity of sorted PKH^-^CD137^+^CD8^+^ cells is 96.1±1.5%.

Supplementary Figure 2: MLR **culture repeats.** Pairwise scatterplots of CD137^+^CD8^+^T cell clonotypes detected in three MLRs done in parallel as triplicates, using HLA-A*02:05 versus 02:01 and C*07:01 versus 14:02 mismatched responder/stimulator cells. Number of shared/total number of clonotypes, % TCR overlap, Morisita’s index and cumulative frequencies of shared clonotypes are provided. The total overlap of the triplicates is 10.2%, with 107/1047 shared clonotypes and cumulative frequencies of 0.58 in MLR1a, 0.66 in MLR1b and 0.27 in MLR1c. Blue dots represent clonotypes that have a statistically significant greater frequency in sample A compared to sample B, red dots represent clonotypes that have a statistically significant greater frequency in sample B compared to sample A, while gray dots represent clonotypes not differing significantly in frequency. Light gray dots clonotypes are excluded from the analyses. Frequency analysis was performed with the (differential abundance tool in ImmunoSEQAnalyzer 3.0).

Supplementary Figure 3: **TNFα and IFNβ** **-induced HLA class I upregulation**. A) HLA-A, B and C cell surface expression measured as mean fluorescence intensity (MFI) on PBMC stimulated (blue dots, mean 125940±46389) or not (red dots, mean 77416±24545) with the cytokines TNFα and IFNβ in 56 anonymous blood donors; mean fold upregulation of 1.65, paired *t*-test p=0.0001 r= 0.84. **B**) HLA–C cell surface expression on PBMC stimulated (blue dots, mean 12872±4794) or not (red dots, mean 6508±3030) with the cytokines TNFα and IFNβ in 32 of the 56 anonymous blood donors; mean fold upregulation of 1.9, paired *t*-test p <0.0001 r=0.92.

Supplementary Figure 4: **Cytokine-induced HLA class I upregulation and its effect on alloreactive CD8^+^ T cell response**. **(A)** HLA-A, B and C cell surface expression (MFI) on PBMC stimulated (blue boxes and dots,mean 178580±81307) or not (red boxes and dots, mean 125239±56683) with the cytokines TNFα and IFNβ (paired *t*-test, p<0.0001). **(B)** % activated CD137^+^CD8^+^ cells retrieved in 20 MLR’s performed with untreated (red boxes,mean 18.5±9.9) or treated (blue boxes, mean 14.59±11.2) stimulating cells shown in A (paired *t*-test, p=0.051).

**Data availability**: DOI will be 10.21417/FSB2020FI ,URL clients.adaptivebiotech.com/pub/ studer-bettens-2020-fi.
